# A novel hydrogel sheet prevents postoperative pancreatic fistula in a rat model

**DOI:** 10.1002/jhbp.867

**Published:** 2020-12-01

**Authors:** Akira Kemmochi, Takafumi Tamura, Yoshio Shimizu, Yohei Owada, Yusuke Ozawa, Katsuji Hisakura, Tatsuya Oda, Yayoi Kawano, Takehisa Hanawa, Nobuhiro Ohkohchi

**Affiliations:** ^1^ Department of Gastrointestinal and Hepato‐Biliary‐Pancreatic Surgery Faculty of Medicine University of Tsukuba Tsukuba Japan; ^2^ Faculty of Pharmaceutical Sciences Tokyo University of Science Noda Japan

**Keywords:** hydrogel, pancreatic fistula, polyvinyl alcohol, postoperative pancreatic fistula

## Abstract

**Aim:**

To evaluate the efficacy of a novel hydrogel sheet in preventing postoperative pancreatic fistula (POPF).

**Background:**

Postoperative pancreatic fistula is a life‐threatening complication. As no study has reported the use of hydrogel sheets in preventing POPF, their effectiveness for that purpose remains unclear.

**Methods:**

A novel hydrogel sheet made of polyvinyl alcohol (PVA) was prepared by the freeze‐thaw method. The pancreatic ducts and surrounding pancreatic parenchyma of rats were transected to induce a pancreatic fistula. Next, the sheet was attached to the transection site. Ascitic fluid amylase and lipase concentrations were measured. Neoveil^®^, a nonwoven polyglycolic acid (PGA) felt, is already clinically used as an absorbable reinforcing material at pancreatic transection sites. Neoveil^®^ was used for comparison, as was VIEWGEL^®^, which is marketed as a wound dressing.

**Results:**

The hydrogel sheet remained in place 48 hours postoperatively. The ascitic amylase concentrations in the control, VIEWGEL^®^‐treated, Neoveil^®^‐treated, and hydrogel‐treated rats, respectively, were 4992.4 ± 5355.7, 1068.4 ± 269.1, 730.2 ± 425.2, and 303.1 ± 240.1 IU/L; the ascitic lipase concentrations were 2279.8 ± 3395.2, 169.5 ± 100.6, 90.4 ± 71.0, and 86.8 ± 59.8 IU/L. The ascitic amylase and lipase levels were significantly lower in the hydrogel group than in the other groups (*P* < .05).

**Conclusions:**

This novel hydrogel sheet effectively prevents pancreatic fistulas and has promising clinical application potential.

## Graphical abstract

***Highlight***

Kemmochi and colleagues developed a novel hydrogel sheet made of polyvinyl alcohol to prevent postoperative pancreatic fistula. Rat pancreases were transected to induce pancreatic fistula, and Viewgel®, Neoveil® or the hydrogel sheet were attached to the transected site. The hydrogel sheet showed a higher preventive effect than existing medical materials.

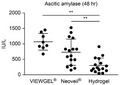

## 1 INTRODUCTION

Although the mortality rate after pancreatectomy has decreased to <5%, the rate of postoperative complications remains high. Postoperative pancreatic fistula (POPF) is a major complication that occurs after pancreatic resection. Several reports have shown that pancreatic fistula occurs in 2%‐45% of patients after pancreaticoduodenectomy and 20%‐50% of patients after pancreatic tail resection.^1^, ^2^, ^3^, ^4^, ^5^, ^6^, ^7^, ^8^, ^9^ POPF can lead to the dissolution of surrounding organs and blood vessels, hemorrhage, and sepsis, all of which eventually result in a prolonged hospital stay, potentially serious and life‐threatening events, or death.^10^, ^11^ Therefore, preventing POPF is the most important clinical issue in pancreatic operations.^12^ Recently, several studies using mesenchymal stem cell sheets to prevent POPF have been reported.^13^, ^14^, ^15^ However, there is a risk of promoting the proliferation of residual tumor cells after pancreatectomy due to (malignant) tumors. There is a report regarding the use of hydrogel sealant to prevent pancreatic fistula, but the sealant is difficult to use.^16^ There have been no reports on the prevention of POPF with PVA hydrogel sheets. Therefore, the aim of this study was to evaluate the efficacy of our newly developed PVA hydrogel sheet for the prevention of POPF.

## 2 METHODS

### 2.1 Preparation of novel hydrogel sheets

**FIGURE 1:**
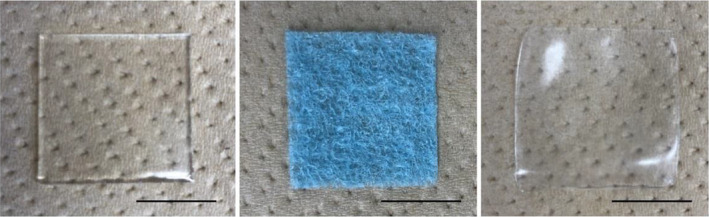
Substances used in this study. VIEWGEL^®^ (A), Neoveil^®^ (B), and the novel hydrogel sheet (C). Scale bar = 10 mm

Polyvinyl alcohol is a synthetic polymer that has high biocompatibility, excellent acid resistance, basic resistance, and chemical resistance. PVA can be hydrogelated and clinically used as a wound dressing in the dermatological field.^17^, ^18^, ^19^, ^20^, ^21^ Exceval^®^ (RS‐2117; Kuraray Corporation), a polyvinyl alcohol, was used to prepare hydrogel sheets in this study. Then, 10% Exceval^®^ was dissolved in ultrapure water. The sheets were dispensed into stainless steel petri dishes, frozen at −20°C for 18 hours and thawed at 35°C for 6 hours. The freeze‐thaw process was performed twice to prepare appropriate hydrogel sheets (Figure 1).

### 2.2 Other materials

VIEWGEL^®^ (TAIHO PHARMA Corporation), is a hydrogel sheet that is marketed as a wound dressing and protector (Figure 1). It is composed of two layers of an absorbent hydrogel and a polyethylene film support. Neoveil^®^ (GUNZE Corporation) is a nonwoven polyglycolic acid (PGA) felt that is clinically used as an absorbable reinforcing material applied to the pancreatic transection site (Figure 1).

### 2.3 Animals

Six‐week‐old male Sprague Dawley (SD) rats, weighing 160‐190 g, were purchased from Charles‐Rivers Laboratories, and acclimated for 1 week before the start of the experiments. So far, there has been no report that there is a sex difference in the occurrence of pancreatic fistula, and as a good‐yielding experimental animal, males were used to exclude the sexual cycle. The rats were housed in a temperature‐, humidity‐, and ventilation‐controlled vivarium and maintained on a 12‐hour light‐dark cycle under specific pathogen‐free conditions. Animal experiments were performed in a humane manner after receiving approval from the Institutional University Experiment Committee of the University of Tsukuba, and the protocols were in accordance with the university's Regulations for Animal Experiments and Fundamental Guidelines for Proper Conduct of Animal Experiment and Related Activities in Academic Research Institutions, under the jurisdiction of the Japanese Ministry of Education, Culture, Sports, Science, and Technology.

### 2.4 Experimental groups

The pancreas and pancreatic duct (splenic duct) were transected by preserving the splenic artery and vein. The rats were divided into four groups as follows: (a) control group (pancreas resected without treatment group; n = 16); (b) VIEWGEL^®^ group (n = 8); (c) Neoveil^®^ group (n = 16); and (d) hydrogel group (n = 16).

### 2.5 Surgical procedure

The rats were anesthetized with machine‐regulated isoflurane. The rats were placed in a supine position. After upper midline laparotomy, the ligaments around the left lobe of the pancreas and spleen were dissected to transect the pancreas and pancreatic duct. The rat pancreatic duct consists of four ducts, including the gastric, duodenal, common, and splenic ducts.^13^ The splenic artery and vein were preserved, and the splenic duct and surrounding pancreatic parenchyma were transected to induce a pancreatic fistula. After transection of the pancreas, VIEWGEL^®^, Neoveil^®^, or the hydrogel sheet was attached to the transection site of the pancreas. The rats were sacrificed 48 hours after the operation. During relaparotomy, the abdominal cavity was rinsed with 5 mL of saline, and ascites was collected for the measurement of amylase and lipase concentrations. Thereafter, pancreatic tissues were collected from each rat.

### 2.6 Analysis of ascitic and serum samples

Amylase and lipase concentrations in ascites were measured using a FUJI DRI‐CHEM 7000V multiple biochemical analyzer (FUJIFILM Corporation).

### 2.7 Histology and immunohistochemistry

Forty‐eight hours after the operation, pancreatic tissues were obtained from each group, fixed with 10% formaldehyde, and embedded in paraffin. Thin sections (3 μm) were prepared and stained with hematoxylin‐eosin (HE). Inflammatory cells in the pancreatic transection site were counted in randomly selected high‐power fields (×400). Immunohistochemical staining for IL‐6 (1:8000, ab9324; Abcam) was performed.

### 2.8 Swelling test

After washing, dried materials were immersed in ultrapure water (10 mL) for 24 hours at room temperature. After wiping excess water from the surface of the materials, the weight of the swollen materials was measured with respect to time (swelling behavior). The degree of swelling at 24 hours was calculated using the following equation:

$$\text{Degree of swelling}=\left(W_{s}‐W_{d}\right)/W_{d}$$

*W*_s_, the weight of the swollen gel (mg) and *W*_d_, the initial weight of the dried gel (mg).

### 2.9 Membrane permeation test

The membrane permeation test was conducted according to the parallel artificial membrane permeation assay (PAMPA).^22^ The Side‐Bi‐Side Cell (PermeGear, Inc.), a blown glass diffusion cell that locates the membrane vertically between the donor chamber and the receptor chamber, both of which are stirred and closed off from the air, was used.

### 2.10 Statistical analysis

All data are expressed as the means ± standard deviation. Statistical analyses were conducted using GraphPad PRISM Version 7.04 (GraphPad Software). The Mann‐Whitney *U* test was used for comparisons between the two groups. One‐way ANOVA was used to compare three or more groups. *P*‐values <.05 were considered significant.

## 3 RESULTS

### 3.1 Development of the POPF model in rats

**FIGURE 2:**
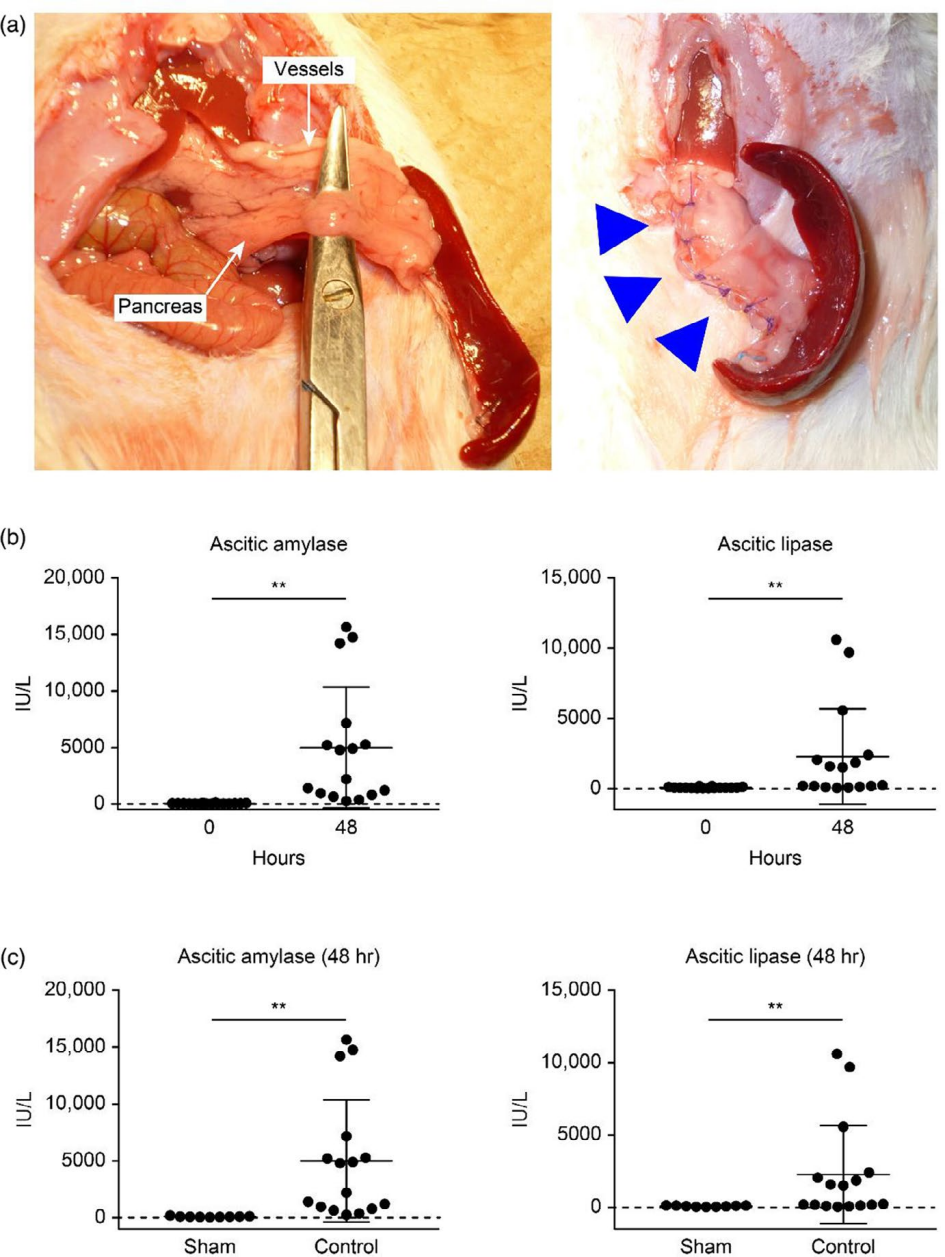
Preparation of the rat pancreatic fistula model and application of the hydrogel sheet. A, After the pancreas and spleen were extracted, the splenic artery and vein were detached and preserved on the dorsal side, and only the pancreatic parenchyma was detached and secured (A‐a). After excision of the pancreas, a hydrogel sheet (arrowhead) was applied and fixed in place with sutures (A‐b). B, Concentrations of pancreatic enzymes in ascitic fluid before pancreatic resection and 48 h after the operation. C, Concentrations of pancreatic enzymes in the ascitic fluid of the sham group and control group 48 h after the operation. The values are expressed as the means ± SDs and were compared using the Mann‐Whitney *U* test. **P < .01

Previous reports have shown that the rat pancreatic duct is composed of four smaller ducts: the gastric duct, duodenal duct, common duct, and splenic duct. The splenic artery and vein were preserved, and the splenic duct and surrounding pancreatic parenchyma were transected to induce a pancreatic fistula as previously reported (Figure 2A‐a). After excision of the pancreas and splenic duct, VIEWGEL^®^, Neoveil^®^, and hydrogel sheets were applied and fixed in place with sutures (Figure 2A‐b). Each substance was applied so as to surround the dissection site and was fixed with 3‐4 single nodular suture stitches. The levels of ascitic amylase and lipase at the time of the operation and 48 hours after the operation were measured (Figure 2B). The pancreatic enzyme level in the ascites 48 hours after the operation was markedly elevated, indicating that a pancreatic fistula had developed. In addition, to rule out the effect of laparotomy on the evaluation of POPF rat model development, a sham operation (the only laparotomy without any transection of the pancreas) was also performed (Figure 2C). In the sham group, no increase in pancreatic enzymes was observed 48 hours after surgery, indicating that laparotomy does not affect the development of pancreatic fistula.

### 3.2 Relaparotomy findings 48 hours after the operation

**FIGURE 3:**
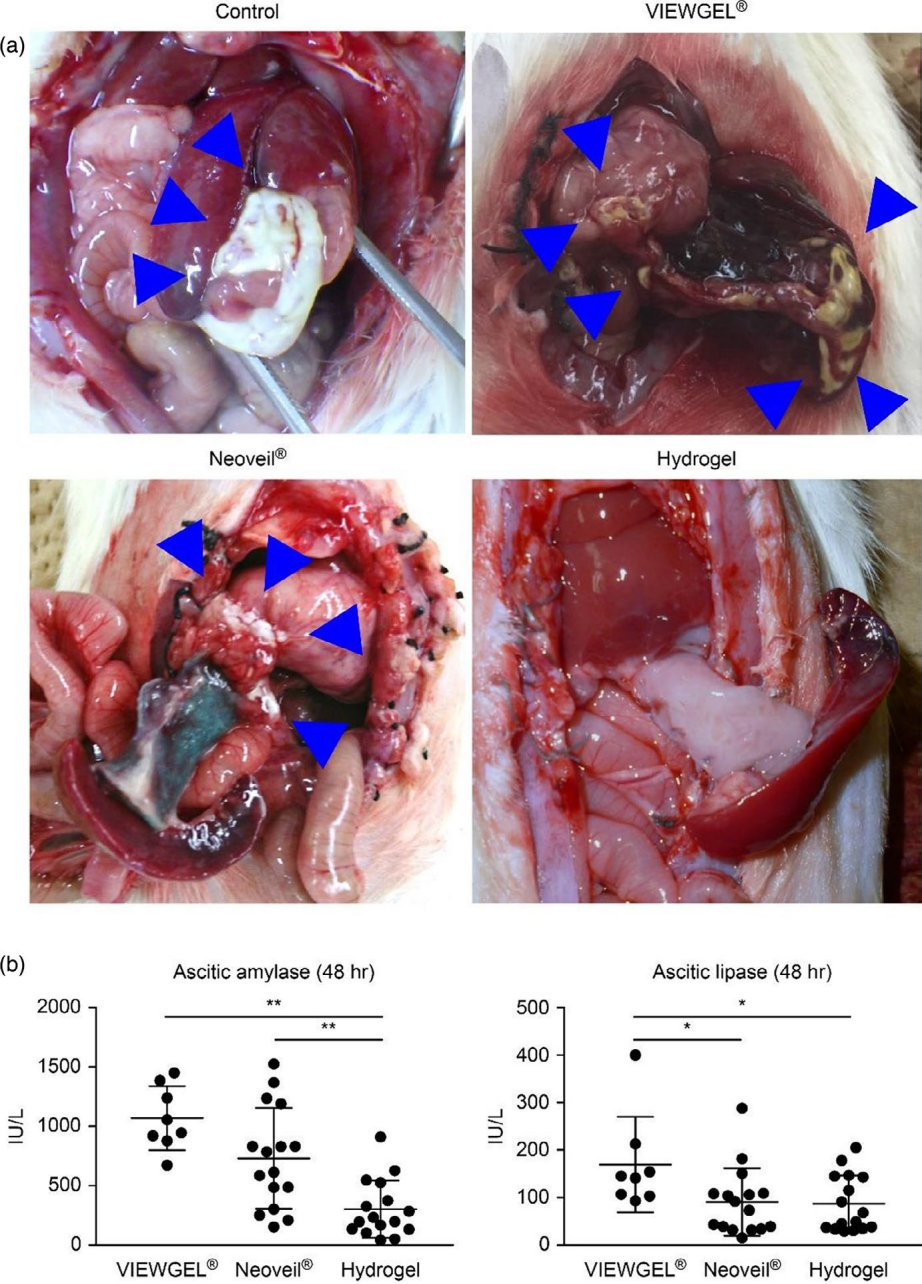
The effects of VIEWGEL^®^, Neoveil^®^, and the hydrogel sheet in a rat pancreatic fistula model. A, Macroscopic findings 48 h after surgery. Saponification is observed in the fatty tissue around the pancreas (except in the region of the hydrogel sheet). B, Concentrations of pancreatic enzymes in ascitic fluid 48 h after surgery. The values are expressed as the means ± SDs and were compared using one‐way ANOVA. *P < .05, **P < .01

The rats were sacrificed 48 hours after the operation. Macroscopically, the fat tissues around the pancreas were saponified in the control, VIEWGEL^®^, and Neoveil^®^ groups but not in the hydrogel group (Figure 3A). The rate of saponification in the control, VIEWGEL^®^, Neoveil^®^, and hydrogel groups was 68.8% (11/16), 75.0% (6/8), 50.0% (8/16), and 0% (0/16), respectively.

### 3.3 Preventive effect of each sheet on pancreatic fistula

The levels of ascitic amylase in the control, VIEWGEL^®^, Neoveil^®^, and hydrogel groups were 4992.4 ± 5355.7, 1068.4 ± 269.1, 730.2 ± 425.2, and 303.1 ± 240.1 IU/L, respectively (Figure 3B). The levels of ascitic lipase were 2279.8 ± 3395.2, 169.5 ± 100.6, 90.4 ± 71.0, and 86.8 ± 59.8 IU/L, respectively. In the hydrogel group, both the levels of amylase and lipase in ascites were significantly lower (*P* < .05).

### 3.4 Immunohistochemistry and haemotoxylin and eosin (HE) staining

**FIGURE 4:**
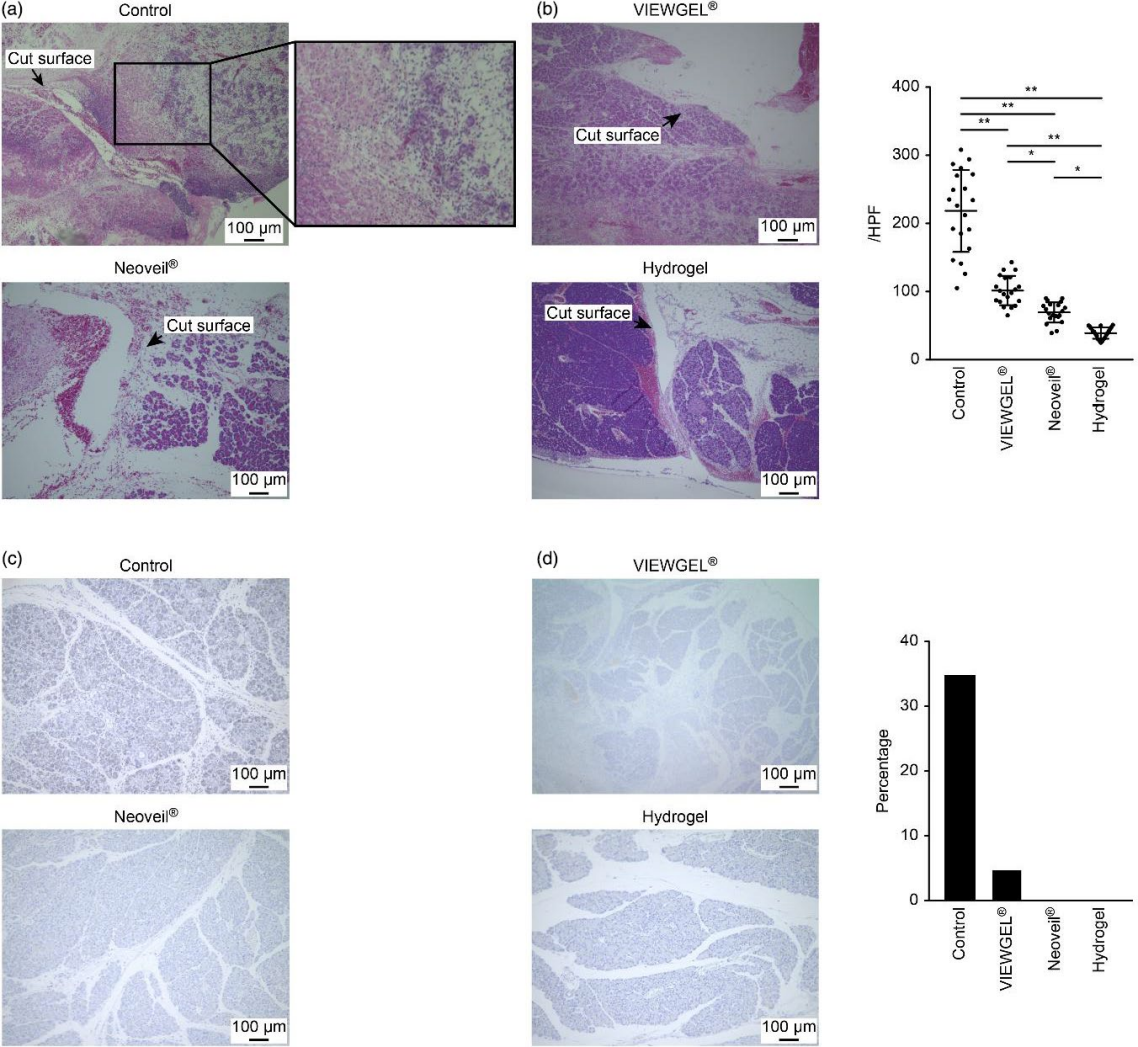
Pathological evaluation and inflammatory cell infiltration of pancreatic tissue 48 h after surgery. Scale bar = 100 µm. A, Histology of the pancreatic transection site assessed by hematoxylin and eosin staining. B, Number of inflammatory cells in 20 high‐power fields. The results are expressed as the means ± SDs and were compared using one‐way ANOVA. *P < .05, **P < .01. C, IL‐6 immunostaining. D, Percentage of area stained positive for IL‐6

HE‐stained sections of the pancreatic transection sites are shown in Figure 4A. Moderate to severe inflammatory cell infiltration was observed in the pancreatic transection site of the control group. Mild acute inflammation was present in the transection site of the VIEWGEL^®^, Neoveil^®^, and hydrogel groups. The number of inflammatory cells in 20 fields of the high‐power field was counted. The number of inflammatory cells in the control, VIEWGEL^®^, Neoveil^®^, and hydrogel groups was 218.4 ± 60.2/HPF, 101.6 ± 21.5/HPF, 69.4 ± 14.8/HPF, and 39.2 ± 8.4/HPF, respectively (Figure 4B). Acinar cells expressing IL‐6 were especially observed in the pancreatic tissues of the control group (Figure 4C,D).

### 3.5 Degree of swelling

**FIGURE 5:**
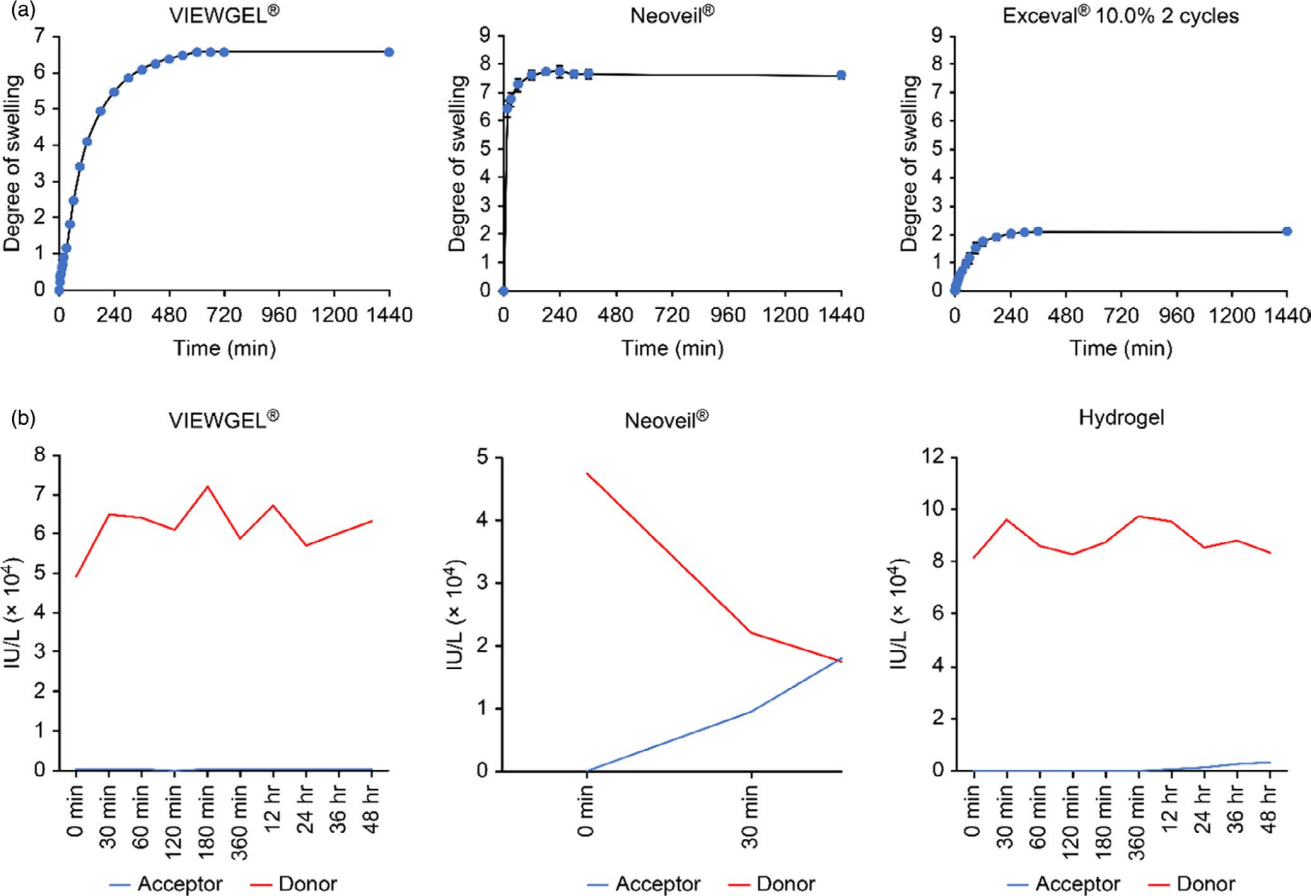
Evaluation of the physical properties of the substances used in this study. A, Swelling test of VIEWGEL^®^, Neoveil^®^, and the hydrogel sheet. The degree of swelling of each material over time was calculated according to an established formula. B, Membrane permeation test of VIEWGEL^®^, Neoveil^®^, and the hydrogel sheet. The red line shows the amylase concentration in the donor chamber, and the blue line shows the amylase concentration in the acceptor chamber. In the VIEWGEL^®^ group, there was no increase in the amylase level in the receptor chamber. In the Neoveil^®^ group, amylase level reached equilibrium between the donor chamber and the receptor chamber. In the hydrogel group, the amylase level in the receptor chamber slowly increased

Figure 5A shows the degree of swelling of the Neoveil^®^ and hydrogel sheets. The degree of swelling became constant 4 hours after the start of the test and with the passage of time. VIEWGEL^®^ and Neoveil^®^ swelled up to approximately seven times their size, while the hydrogel sheet swelled up to approximately two times its size.

### 3.6 Behavior of amylase

Membrane permeation tests were performed to determine how amylase behaves with VIEWGEL^®^, Neoveil^®^, and hydrogels (Figure 5B). In the VIEWGEL^®^ group, there was no increase in the amylase level in the receptor chamber due to the presence of a polyethylene film in the outer layer. Neoveil^®^ is a nonwoven polyglycolic acid felt that has a low ability to block liquids. In the Neoveil^®^ group, amylase values reached equilibrium in the donor chamber and the receptor chamber in approximately 40 minutes. In the hydrogel group, the amylase level in the receptor chamber was slowly increased, and at 48 hours, the level increased to 3.5% of the donor chamber amylase level.

## 4 DISCUSSION

Although there have been significant improvements in pancreatic surgery in recent years, pancreatic fistula still occurs at a high rate.^1^, ^2^, ^3^, ^4^, ^5^, ^6^, ^7^, ^8^, ^9^ It is ideal for pancreatic surgeons to achieve a zero incidence of pancreatic fistula, but improvements in surgical techniques and perioperative management have almost reached their limits. POPF is a life‐threatening complication that significantly extends hospital stays and increases medical costs.^23^ Currently, many operative methods, new devices, and medications have been used in an attempt to prevent POPF.^24^, ^25^, ^26^ However, there is still no definitive way to prevent this adverse event. Typical substances for POPF prevention are fibrin glue and polyglycolic acid felts.^27^, ^28^, ^29^, ^30^, ^31^, ^32^ Fibrin glue sealant is used in the pancreatic anastomosis and on the dissection surfaces, and this step may reduce the occurrence of pancreatic fistulas. However, a recent large‐scale meta‐analysis that evaluated the effect of fibrin glue on pancreatic surgery reported no significant effect.^27^, ^28^, ^29^, ^30^, ^31^ A nonwoven polyglycolic acid (PGA) felt (Neoveil^®^) has been reported to reduce the incidence of pancreatic fistula but has not yet yielded significant improvement.^32^ These results indicate that the current material in use is not sufficient to prevent pancreatic fistulas and that new ideas are needed. To prevent POPF, attempts have been made to completely seal the anastomosis and the dissection surface of the pancreas.^24^, ^25^, ^26^, ^27^, ^28^, ^29^, ^30^, ^31^, ^32^ These results are inadequate, and there is a need for substances that have the ability to absorb and retain leaking pancreatic juice. There are some reports of the use of mesenchymal stem cell sheets^14^, ^15^; however, when employed in cancer surgery, they may be a risk factor for recurrence of the anastomosis and stumps, and clinical application is difficult. We focused on PVA, which is already used in the dermatology field and is established to be safe.^17^, ^18^, ^19^, ^20^, ^21^ A freeze‐thaw method that can be employed to adjust the physical properties was selected, using a physical crosslinking method that does not involve harmful crosslinking chemicals.^33^, ^34^, ^35^, ^36^ Thus, a novel hydrogel sheet with high flexibility, high tensile strength, low elasticity, and low swellability was developed. The aim of this study was to evaluate the efficacy of novel therapy for the prevention of POPF using newly developed hydrogel sheets. As a result, this novel hydrogel sheet was effective for preventing pancreatic fistulas.

To examine the preventive effect of the hydrogel sheet against pancreatic fistula, it is indispensable to create a model animal that produces a pancreatic fistula. Tanaka et al^13^ reported that among procedures on the four sections of the rat pancreatic duct, dissection of the splenic duct induced the optimal pancreatic fistula in a model animal. In this study, we preserved the splenic artery and vein of the rat and created a pancreatic fistula from the splenic duct by excising the pancreatic parenchyma. A significant increase was observed in pancreatic enzyme levels in ascites before and 48 hours after surgery. Compared with the corresponding levels in the sham group at 48 hours postoperatively, pancreatic enzymes in the ascites were significantly higher, which was appropriate as a pancreatic fistula model. In this study, there was a significant difference in pancreatic enzyme levels in the ascites between the control group and the VIEWGEL^®^, Neoveil^®^, and hydrogel groups. These results indicate that hydrogel sheets have the potential to prevent pancreatic fistula most effectively. Although the present model did not fully reflect clinical situations such as intra‐abdominal inflammation, sepsis, and abscesses, we found that inflammatory cell infiltration was lower in the hydrogel sheet group than in either of the other groups. Therefore, it can be stated that this hydrogel sheet is suitable for the prevention of pancreatic fistula.

When exposed to pancreatic enzymes, adipocyte cell membranes are damaged, adipocytes release soap‐like triglycerides, and adipose tissue becomes white.^37^ The result is saponification, which is an index for the clinical confirmation of pancreatic fistula.^38^ In this study, saponification was observed in the control, VIEWGEL^®^, and Neoveil^®^ groups 48 hours postoperatively, but not in the hydrogel group. Consequently, these results suggested that the hydrogel sheet can prevent pancreatic fistula formation. Inflammation is also an important factor that reflects a pancreatic fistula.^39^ The number of inflammatory cells in the resected area of the excised pancreatic tissue was lowest in the hydrogel group, suggesting that local inflammatory responses were also suppressed. The fact that saponification did not occur and the fact that inflammation was suppressed demonstrated the effectiveness of the hydrogel sheet.

The characteristics of the hydrogel sheet that we developed are high flexibility, high tensile strength, low elasticity, and low swellability. The low swellability was particularly effective in preventing pancreatic fistula formation. Both VIEWGEL^®^ and Neoveil^®^ used as comparative substances showed excessive swelling, while that of the hydrogel sheet was optimal. Low swelling is desirable in the medical material used to prevent pancreatic fistula. The reason is that if the substance is excessively swollen, the volume will swell and there is a risk of pressure or injury to surrounding tissues. Hydrogels generally show a high degree of swelling when hydrated. The hydrogel sheet we have developed in this study is a special hydrogel that has a strong crosslinked structure and does not swell excessively, so the effect on surrounding tissues should be minimal. The other is the maintenance of form. By applying hydrogel sheet to the pancreatic dissection site, we expect the effect of physically containing the pancreatic fistula. When the rats in each group were reopened 48 hours after the operation, VIEWGEL^®^ and Neoveil^®^ were unable to maintain their morphology due to excessive swelling, and some individuals had poor adhesion to the pancreatic dissection site. On the other hand, excessive swelling did not occur in the hydrogel group, and adhesion to the pancreatic dissection site was maintained as it was at the time of the first surgery. According to these results, VIEWGEL^®^ and Neoveil^®^ are considered to be inferior to hydrogel sheet in preventing pancreatic fistulas. Therefore, due to the low swelling, the hydrogel sheet was able to maintain firm adhesion to the pancreatic dissection site, and it is thought that it exerted a high pancreatic fistula prevention effect without causing pressure or injury to the surrounding tissues.

The membrane permeation test suggested that the hydrogel sheet tends to retain pancreatic juice for a long time and does not leak it into the surroundings. VIEWGEL^®^ is impervious to liquids due to the presence of the outermost polyethylene film. Theoretically, if it is sealed, pancreatic juice should not leak into the surroundings, but the experimental results showed pancreatic fistulas. This is because VIEWGEL^®^ has high elasticity and high swelling properties, which are the opposite properties of the hydrogel sheet. These features are disadvantageous for maintaining adhesion to organs and maintaining their shape in the abdominal cavity. Neoveil^®^ is considered to have almost no ability to hold or block liquid because it reaches equilibrium in approximately 40 minutes in the membrane permeation test. Pancreatic alpha‐amylase has a half‐life of 120 minutes.^40^ In the membrane permeation test, it is estimated that the hydrogel sheet itself retains pancreatic juice for approximately 24 hours and does not leak it into the surroundings. Meanwhile, the enzyme activity of amylase also decreases, and leaked amylase may not affect the surrounding tissue. To date, it has been considered that the best way to prevent pancreatic fistula is to completely seal the anastomosis and the dissection surface of the pancreas.^24^, ^25^, ^26^, ^27^, ^28^, ^29^, ^30^, ^31^, ^32^ However, complete sealing is impossible in practice, and pancreatic fistulas have occurred even when a situation close to that described above is created. The hydrogel sheet as examined in this study is expected to minimize the influence on surrounding tissues by retaining the leaked pancreatic juice. This is a new concept of pancreatic fistula prevention and may be the most effective means.

### 4.1 Limitation

Although pancreatic fistula could be evaluated by pancreatic enzymes in ascites, we have not been able to evaluate complications such as abscess formation and pseudoaneurysm formation caused by pancreatic fistula. If image examination such as computed tomography (CT) can be performed instead of laparotomy, it may be possible to make an objective evaluation by fluid retention in the abdominal cavity and increase in blood vessel diameter.

In addition, since the morphology of the pancreas differs between rats and humans, we believe that experiments with large animals such as pigs, which are similar to the morphology of human organs, will be necessary in the future.

## 5 CONCLUSION

A novel hydrogel sheet composed of Exceval® has significant potential for preventing pancreatic fistulas. The effect of this hydrogel sheet has good compatibility with the drainage tube already used in clinical practice, and a synergistic effect is expected. We plan to test our experimental design in a porcine model to further determine its clinical potential.

## CONFLICT OF INTEREST

Authors declare no conflict of interest for this article.

## References

[1] Bassi C , Dervenis C , Butturini G , Fingerhut A , Yeo C , Izbicki J , et al. Postoperative pancreatic fistula: an international study group (ISGPF) definition. Surgery. 2005;138:8‐13. 16003309 10.1016/j.surg.2005.05.001

[2] Büchler MW , Friess H , Wagner M , Kulli C , Wagener V , Z'Graggen K . Pancreatic fistula after pancreatic head resection. Br J Surg. 2000;87:883‐9. 10931023 10.1046/j.1365-2168.2000.01465.x

[3] Fahy BN , Frey CF , Ho HS , Beckett L , Bold RJ . Morbidity, mortality, and technical factors of distal pancreatectomy. Am J Surg. 2002;183:237‐41. 11943118 10.1016/s0002-9610(02)00790-0

[4] Christein JD , Kendrick ML , Iqbal CW , Nagorney DM , Farnell MB . Distal pancreatectomy for resectable adenocarcinoma of the body and tail of the pancreas. J Gastrointest Surg. 2005;9:922‐7. 16137585 10.1016/j.gassur.2005.04.008

[5] Knaebel HP , Diener MK , Wente MN , Büchler MW , Seiler CM . Systematic review and meta‐analysis of technique for closure of the pancreatic remnant after distal pancreatectomy. Br J Surg. 2005;92:539‐46. 15852419 10.1002/bjs.5000

[6] Velanovich V . Case‐control comparison of laparoscopic versus open distal pancreatectomy. J Gastrointest Surg. 2006;10:95‐8. 16368497 10.1016/j.gassur.2005.08.009

[7] Akamatsu N , Sugawara Y , Komagome M , Shin N , Cho N , Ishida T , et al. Risk factors for postoperative pancreatic fistula after pancreaticoduodenectomy: the significance of the ratio of the main pancreatic duct to the pancreas body as a predictor of leakage. J Hepatobiliary Pancreat Sci. 2010;17:322‐8. 20464562 10.1007/s00534-009-0248-6

[8] Schoellhammer HF , Fong Y , Gagandeep S . Techniques for prevention of pancreatic leak after pancreatectomy. Hepatobiliary Surg Nutr. 2014;3:276‐87. 25392839 10.3978/j.issn.2304-3881.2014.08.08 PMC4207840

[9] Liang S , Hameed U , Jayaraman S . Laparoscopic pancreatectomy: indications and outcomes. World J Gastroenterol. 2014;20:14246‐54. 25339811 10.3748/wjg.v20.i39.14246 PMC4202353

[10] van Berge Henegouwen MI , De Wit LT , Van Gulik TM , Obertop H , Gouma DJ . Incidence, risk factors, and treatment of pancreatic leakage after pancreaticoduodenectomy: drainage versus resection of the pancreatic remnant. J Am Coll Surg. 1997;185:18‐24. 9208956 10.1016/s1072-7515(97)00007-0

[11] Butturini G , Daskalaki D , Molinari E , Scopelliti F , Casarotto A , Bassi C . Pancreatic fistula: definition and current problems. J Hepatobiliary Pancreat Surg. 2008;15:247‐51. 18535760 10.1007/s00534-007-1301-y

[12] Uzbas F , May ID , Parisi AM , Thompson SK , Kaya A , Perkins AD , et al. Molecular physiognomies and applications of adipose‐derived stem cells. Stem Cell Rev Rep. 2015;11:298‐308. 25504377 10.1007/s12015-014-9578-0

[13] Tanaka T , Kuroki T , Adachi T , Ono S , Kitasato A , Hirabaru M , et al. Development of a novel rat model with pancreatic fistula and the prevention of this complication using tissue‐engineered myoblast sheets. J Gastroenterol. 2013;48:1081‐9. 23179607 10.1007/s00535-012-0706-9

[14] Kaneko H , Kokuryo T , Yokoyama Y , Yamaguchi J , Yamamoto T , Shibata R , et al. Novel therapy for pancreatic fistula using adipose‐derived stem cell sheets treated with mannose. Surgery. 2017;161:1561‐9. 28143661 10.1016/j.surg.2016.12.022

[15] Kim SR , Yi HJ , Lee YN , Park JY , Hoffman RM , Okano T , et al. Engineered mesenchymal stem‐cell‐sheets patches prevents postoperative pancreatic leakage in a rat model. Sci Rep. 2018;8:360. 29321630 10.1038/s41598-017-18490-9 PMC5762914

[16] Balcom JHT , Keck T , Warshaw AL , Graeme‐Cook F , Fernández‐del CC . Prevention of pancreatic fistula with a new synthetic, absorbable sealant: evaluation in a dog model. J Am Coll Surg. 2002;195:490‐6. 12375754 10.1016/s1072-7515(02)01313-3

[17] Kamoun EA , Kenawy ES , Chen X . A review on polymeric hydrogel membranes for wound dressing applications: PVA‐based hydrogel dressings. J Adv Res. 2017;8:217‐33. 28239493 10.1016/j.jare.2017.01.005 PMC5315442

[18] Suhaeri M , Noh MH , Moon JH , Kim IG , Oh SJ , Ha SS , et al. Novel skin patch combining human fibroblast‐derived matrix and ciprofloxacin for infected wound healing. Theranostics. 2018;8:5025‐38. 30429884 10.7150/thno.26837 PMC6217057

[19] Fan L , Yang H , Yang J , Peng M , Hu J . Preparation and characterization of chitosan/gelatin/PVA hydrogel for wound dressings. Carbohydr Polym. 2016;146:427‐34. 27112893 10.1016/j.carbpol.2016.03.002

[20] Baghaie S , Khorasani MT , Zarrabi A , Moshtaghian J . Wound healing properties of PVA/starch/chitosan hydrogel membranes with nano Zinc oxide as antibacterial wound dressing material. J Biomater Sci Polym Ed. 2017;28:2220‐41. 28988526 10.1080/09205063.2017.1390383

[21] Ahmed AS , Mandal UK , Taher M , Susanti D , Jaffri JM . PVA‐PEG physically cross‐linked hydrogel film as a wound dressing: experimental design and optimization. Pharm Dev Technol. 2018;23:751‐60. 28378604 10.1080/10837450.2017.1295067

[22] Buckley ST , Fischer SM , Fricker G , Brandl M . *In vitro* models to evaluate the permeability of poorly soluble drug entities: challenges and perspectives. Eur J Pharm Sci. 2012;45:235‐50. 22178532 10.1016/j.ejps.2011.12.007

[23] Dindo D , Demartines N , Clavien PA . Classification of surgical complications: a new proposal with evaluation in a cohort of 6336 patients and results of a survey. Ann Surg. 2004;240:205‐13. 15273542 10.1097/01.sla.0000133083.54934.ae PMC1360123

[24] Choi SB , Lee JS , Kim WB , Song TJ , Suh SO , Choi SY . Efficacy of the omental roll‐up technique in pancreaticojejunostomy as a strategy to prevent pancreatic fistula after pancreaticoduodenectomy. Arch Surg. 2012;147:145‐50. 22351908 10.1001/archsurg.2011.865

[25] Matsusue S , Takeda H , Nakamura Y , Nishimura S , Koizumi S . A prospective analysis of the factors influencing pancreaticojejunostomy performed using a single method, in 100 consecutive pancreaticoduodenectomies. Surg Today. 1998;28:719‐26. 9697265 10.1007/BF02484618

[26] Kuroki T , Tajima Y , Tsutsumi R , Tsuneoka N , Fukuda K , Haraguchi M , et al. Gastric wall‐covering method for the prevention of pancreatic fistula after pancreatic resection. Hepatogastroenterology. 2007;54:935‐6. 17591096

[27] Kram HB , Clark SR , Ocampo HP , Yamaguchi MA , Shoemaker WC . Fibrin glue sealing of pancreatic injuries, resections, and anastomoses. Am J Surg. 1991;161(4):479‐81; discussion 82. 2035768 10.1016/0002-9610(91)91116-z

[28] D'Andrea AA , Costantino V , Sperti C , Pedrazzoli S . Human fibrin sealant in pancreatic surgery: it is useful in preventing fistulas? A prospective randomized study. Ital J Gastroenterol. 1994;26:283‐6. 7949264

[29] Suzuki Y , Kuroda Y , Morita A , Fujino Y , Tanioka Y , Kawamura T , et al. Fibrin glue sealing for the prevention of pancreatic fistulas following distal pancreatectomy. Arch Surg. 1995;130:952‐5. 7661678 10.1001/archsurg.1995.01430090038015

[30] Suc B , Msika S , Fingerhut A , Fourtanier G , Hay JM , Holmières F , et al. Temporary fibrin glue occlusion of the main pancreatic duct in the prevention of intra‐abdominal complications after pancreatic resection: prospective randomized trial. Ann Surg. 2003;237:57‐65. 12496531 10.1097/00000658-200301000-00009 PMC1513966

[31] Orci LA , Oldani G , Berney T , Andres A , Mentha G , Morel P , et al. Systematic review and meta‐analysis of fibrin sealants for patients undergoing pancreatic resection. HPB (Oxford). 2014;16:3‐11. 23461684 10.1111/hpb.12064 PMC3892308

[32] Jang JY , Shin YC , Han Y , Park JS , Han HS , Hwang HK , et al. Effect of polyglycolic acid mesh for prevention of pancreatic fistula following distal pancreatectomy: a randomized clinical trial. JAMA Surg. 2017;152:150‐5. 27784046 10.1001/jamasurg.2016.3644

[33] Akhtar MF , Hanif M , Ranjha NM . Methods of synthesis of hydrogels. A review. Saudi Pharm J. 2016;24:554‐9. 27752227 10.1016/j.jsps.2015.03.022 PMC5059832

[34] Hennink WE , van Nostrum CF . Novel crosslinking methods to design hydrogels. Adv Drug Deliv Rev. 2002;54:13‐36. 11755704 10.1016/s0169-409x(01)00240-x

[35] Zhang H , Zhang F , Wu J . Physically crosslinked hydrogels from polysaccharides prepared by freeze–thaw technique. React Funct Polym. 2013;73:923‐8.

[36] Holloway JL , Lowman AM , Palmese GR . The role of crystallization and phase separation in the formation of physically cross‐linked PVA hydrogels. Soft Matter. 2013;9:826‐33.

[37] Hafezi‐Nejad N , Fishman EK , Zaheer A . Imaging of post‐operative pancreas and complications after pancreatic adenocarcinoma resection. Abdom Radiol (NY). 2018;43:476‐88. 29094173 10.1007/s00261-017-1378-y

[38] Chiba N , Abe Y , Yokozuka K , Hikita K , Kobayashi T , Sano T , et al. Surgical technique of pancreatic parenchyma transection‐delayed approach (PPTDA) in hepatopancreatoduodenectomy for hilar cholangiocarcinoma. J Gastrointest Surg. 2019;23:613‐6. 30187328 10.1007/s11605-018-3923-6

[39] De Schryver N , Wittebole X , Hubert C , Gigot JF , Laterre PF , Castanares‐Zapatero D . Early hyperlactatemia predicts pancreatic fistula after surgery. BMC Anesthesiol. 2015;15:109. 26215981 10.1186/s12871-015-0093-x PMC4517345

[40] Apple F , Benson P , Preese L , Eastep S , Bilodeau L , Heiler G . Lipase and pancreatic amylase activities in tissues and in patients with hyperamylasemia. Am J Clin Pathol. 1991;96:610‐4. 1719798 10.1093/ajcp/96.5.610

